# THE EVOLUTION OF NURSING HOME COMMUNICATION WITH RESIDENT FAMILIES DURING THE COVID-19 PANDEMIC

**DOI:** 10.1093/geroni/igad104.3639

**Published:** 2023-12-21

**Authors:** Courtney Hawes, Joan Brazier, Amy Meehan, Emily Gabois

**Affiliations:** Brown University School of Public Health, Providence, Rhode Island, United States; Brown University School of Public Health, Providence, Rhode Island, United States; Brown University School of Public Health, Providence, Rhode Island, United States; Brown University School of Public Health, Providence, Rhode Island, United States

## Abstract

Nursing homes experienced numerous challenges resulting from the COVID-19 pandemic. Among these challenges was ensuring appropriate and effective communication with friends and family members of residents, particularly in light of visitation restrictions. Using qualitative data from 156 in-depth, semi-structured interviews with administrators of 40 nursing homes across the US from July 2020 through December 2021, challenges and solutions to communicating effectively with resident families are characterized. Findings include three themes: 1. Administrators identified best practices regarding the frequency of communicating with families. Particularly early in the pandemic and when visitation was restricted, families expressed a desire for very frequent updates regarding resident well-being and COVID-19. Administrators reported the challenges of balancing family expectations regarding information with their capacity to meet those expectations. 2. Administrators discussed different modes of communication used with families. Existing systems of mass-communication were expanded and new processes were developed. 3. Administrators described challenges in determining the appropriate content to communicate and navigating changing policies. While nursing home staff were required to share data on COVID-19 outbreaks, administrators shared lessons learned regarding specificity of information to be shared, and how they balanced required infection control information with pleasant updates regarding residents and their activities. Strategies identified by administrators may be informative for efforts to promote effective communication for both general day-to-day updates and for future emergency or pandemic crises.

